# Recent Developments in Layer‐by‐Layer Assembly for Drug Delivery and Tissue Engineering Applications

**DOI:** 10.1002/adhm.202302713

**Published:** 2024-01-07

**Authors:** João Borges, Jinfeng Zeng, Xi Qiu Liu, Hao Chang, Claire Monge, Charlotte Garot, Ke‐feng Ren, Paul Machillot, Nihal E. Vrana, Philippe Lavalle, Takami Akagi, Michiya Matsusaki, Jian Ji, Mitsuru Akashi, João F. Mano, Varvara Gribova, Catherine Picart

**Affiliations:** ^1^ CICECO – Aveiro Institute of Materials Department of Chemistry University of Aveiro Campus Universitário de Santiago Aveiro 3810‐193 Portugal; ^2^ Division of Applied Chemistry Graduate School of Engineering Osaka University 2‐1 Yamadaoka Suita Osaka 565–0871 Japan; ^3^ School of Pharmacy Tongji Medical College Huazhong University of Science and Technology Wuhan 430030 China; ^4^ Hangzhou Institute of Medicine Chinese Academy of Sciences Hangzhou Zhejiang 310022 China; ^5^ Laboratory of Tissue Biology and Therapeutic Engineering (LBTI) UMR5305 CNRS/Universite Claude Bernard Lyon 1 7 Passage du Vercors Lyon 69367 France; ^6^ Université de Grenoble Alpes CEA INSERM U1292 Biosanté CNRS EMR 5000 Biomimetism and Regenerative Medicine (BRM) 17 avenue des Martyrs Grenoble F‐38054 France; ^7^ Department of Polymer Science and Engineering Zhejiang University Hangzhou 310027 China; ^8^ SPARTHA Medical 1 Rue Eugène Boeckel Strasbourg 67000 France; ^9^ Institut National de la Santé et de la Recherche Médicale Inserm UMR_S 1121 Biomaterials and Bioengineering Centre de Recherche en Biomédecine de Strasbourg 1 rue Eugène Boeckel Strasbourg 67000 France; ^10^ Université de Strasbourg Faculté de Chirurgie Dentaire 1 place de l'Hôpital Strasbourg 67000 France; ^11^ Building Block Science Joint Research Chair Graduate School of Frontier Biosciences Osaka University 1–3 Yamadaoka Suita Osaka 565–0871 Japan

**Keywords:** biomaterials, cell signaling, drug delivery, growth factors, layer‐by‐layer, regenerative medicine, tissue engineering

## Abstract

Surfaces with biological functionalities are of great interest for biomaterials, tissue engineering, biophysics, and for controlling biological processes. The layer‐by‐layer (LbL) assembly is a highly versatile methodology introduced 30 years ago, which consists of assembling complementary polyelectrolytes or biomolecules in a stepwise manner to form thin self‐assembled films. In view of its simplicity, compatibility with biological molecules, and adaptability to any kind of supporting material carrier, this technology has undergone major developments over the past decades. Specific applications have emerged in different biomedical fields owing to the possibility to load or immobilize biomolecules with preserved bioactivity, to use an extremely broad range of biomolecules and supporting carriers, and to modify the film's mechanical properties via crosslinking. In this review, the focus is on the recent developments regarding LbL films formed as 2D or 3D objects for applications in drug delivery and tissue engineering. Possible applications in the fields of vaccinology, 3D biomimetic tissue models, as well as bone and cardiovascular tissue engineering are highlighted. In addition, the most recent technological developments in the field of film construction, such as high‐content liquid handling or machine learning, which are expected to open new perspectives in the future developments of LbL, are presented.

## Graphical abstract

Layer‐by‐layer (LbL) films represent a powerful approach for surface functionalization and for the production of free‐standing objects. This article reviews recent applications in drug delivery and tissue engineering, coming closer to clinical applications. New methods for LbL deposition and artificial intelligence will foster LbL developments in the near future.

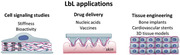

## 1 Introduction

Over the last 30 years, the layer‐by‐layer (LbL) assembly method has proven to be a simple, inexpensive, robust, flexible, and highly versatile bottom‐up surface engineering approach to conformally coat various surface types under mild conditions, from metallic implants to living cells. The LbL technology can be used to engineer a wide variety of highly organized and ordered multilayered nano‐architectures with precisely‐tailored compositions and structures, physicochemical and biochemical properties, as well as (multi)functionalities. This technology offers numerous opportunities for innovation that have turned it into a well‐established and prominent nanofabrication approach in different application fields, including the biomedical field.^[^ ^1^, ^2^, ^3^, ^4^ ^]^ Its simplicity relies on the sequential exposure of various substrates, regardless of size, shape, and surface chemistry, to at least two solutions of multivalent molecules exhibiting complementary interactions, resulting in the formation of a nanometric multilayered structure. Historically, the most popular mechanism for manufacturing nanostructured multilayered assemblies is based on electrostatic interactions, used in the pioneering work of Iler, Decher, Hong, Schmitt, Lvov, and Möhwald in order to assemble oppositely charged colloidal particles,^[^ ^5^ ^]^ bipolar amphiphiles,^[^ ^6^ ^]^ polyelectrolytes,^[^ ^7^, ^8^ ^]^ or a combination thereof^[^ ^9^ ^]^ onto charged planar substrates via the immersive technology.

However, over the last two decades, the LbL technology has received tremendous interest and has evolved rapidly. Its high versatility and remarkable power are also unlocked by demonstrating that a wide array of non‐electrostatic intermolecular forces can also effectively drive, increase the stability, and tailor the multilayered film growth and properties. These forces include hydrophobic, host‐guest, charge‐transfer, coordination chemistry, and biochemically specific interactions, as well as stereo‐complexation, covalent bonding and hydrogen bonding, and a combination thereof.^[^ ^10^ ^]^

Thus, an unlimited number and variety of charged and uncharged building blocks, from different sources and nature, including polymers, proteins, peptides, enzymes, nucleic acids, viruses, living cells, nanoparticles, carbon nanotubes, clays, and dyes, among others, can be alternatively assembled into multicomponent multilayered films denoting new capabilities and multifunctionalities.^[^ ^11^ ^]^ In particular, as the assembly process can occur under mild conditions in entirely aqueous solutions avoiding harmful solvents, high temperatures, extreme ionic strengths, or pH values, this turns LbL assembly into an environmentally friendly surface engineering nanotechnology.

Besides the intrinsic features of the LbL ingredients, including molecule concentration, molecular weight, and charge density, the experimental assembling conditions, which include solution pH, ionic strength, temperature, as well as many adsorbed layers and assembly times, can be carefully chosen. This enables fine‐tuning the overall structure, properties, and functions of the assembled multilayered nanostructures and, ultimately, their performance.^[^ ^10^, ^12^ ^]^

Moreover, the multilayers can be assembled on a given substrate through a variety of assembly methodologies, which determine the processing and material properties, as well as the practical end‐use of the as‐developed LbL architectures. LbL assembly methods include immersion, which is the most widely reported, but also the spin‐coating and spraying methodologies, which have been mainly implemented to generate cost‐effective and faster multilayers, in view of industrial mass production.^[^ ^10^, ^13^ ^]^

More recently, other promising assembly methodologies, including electromagnetic‐ and microfluidic‐driven assembly,^[^ ^13^ ^]^ perfusion,^[^ ^14^ ^]^ high‐gravity field,^[^ ^15^ ^]^ inkjet‐printing,^[^ ^16^ ^]^ as well as LbL deposition on particles,^[^ ^17^, ^18^, ^19^ ^]^ have been also reported. This extends the typical fabrication of multilayered assemblies from flat 2D planar substrates to more convoluted 3D surfaces, for example, colloidal particles, cylindrical structures, porous constructs, microorganisms, or even living cells.

Such panoply of assembly methodologies illustrates the numerous technological developments that the LbL technology has undergone over the last decades. Given this context, LbL assembly is a very appealing technology for addressing a plethora of biomedical and biotechnological applications, owing to its intrinsic capacity to integrate, protect, and preserve the native structure, physicochemical properties, and functions, as well as the biological activity of bioactive molecules and therapeutic agents.^[^ ^3^, ^20^, ^21^, ^22^, ^23^, ^24^ ^]^

**Figure 1:**
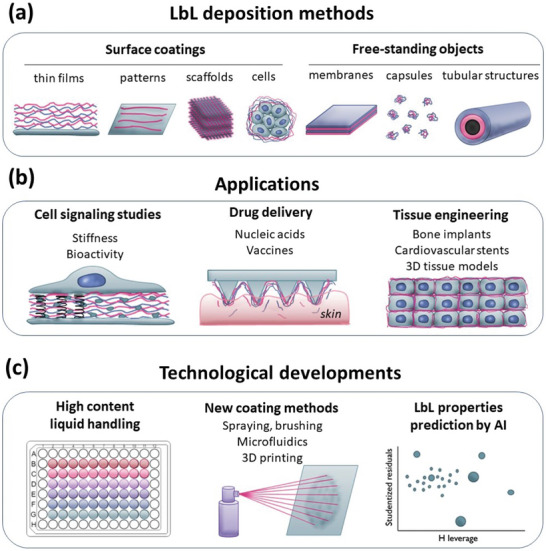
Schematic representation of the review. a) Layer‐by‐layer (LbL) is a powerful method to create different 2D and 3D architectures. b) Examples of LbL biomedical applications. c) New technological developments to make LbL development faster and cheaper. AI: artificial intelligence.

In this review, we provide an overview of the most recent developments regarding LbL assemblies applied to the biomedical field, especially in tissue engineering and drug delivery (**Figure** **1**). Since this field is very broad, we focus on tissue regeneration and tissue model construction, without reviewing the challenges of bacterial and fungal infections. Although these infections can seriously affect the biomaterial functionalities and can even result in failure, we consider this to be a problem on its own, and several reviews have already focused on this aspect.^[^ ^25^ ^]^ Herein, we first discuss the new LbL assembly types, including the coating of complex 2D surfaces or advanced 3D systems for the engineering of various tissue types, from bone to cardiac tissues (Section 2). Next, we present different LbL film applications. The third section focuses on using LbL films to mimic some aspects of the cellular microenvironment, which greatly impact both tissue organization and function. By reproducing several biophysical cues, notably stiffness and the presence of bioactive molecules, LbL can be employed for cell signaling studies. In the fourth section, we provide information about new developments in the field of LbL assembly for nucleic acids and vaccine delivery. In the fifth section, we present selected applications of tissue engineering, especially for bone implants, cardiovascular devices, and 3D tissue models. In the sixth section, we disclose recent technological developments, including the preparation of films using automatic liquid handling robots, which allow for carrying out high‐content studies. Moreover, new coating methods, including spraying, brushing, microfluidics, and 3D printing, and LbL properties predicted in silico by artificial intelligence (AI) are covered. All these approaches allow for the development of multifunctional LbL films in a faster and cost‐effective manner, which is an asset for boosting their application in healthcare. We conclude with future perspectives in the field, which are pushing the boundaries of LbL assembly technology to facilitate its clinical translation and commercialization in the near future.

## 2 LbL Architectures

Owing to the LbL assembly technology's intrinsic capacity to reproduce with high fidelity the topographical features and geometry of templates, mostly in biologically favorable environments, this technology has been widely employed to develop suitable soft matter‐based multidimensional multilayered nanodevices with a high degree of potential biomedical applicability and clinical translation. To date, numerous original research articles and review papers have disclosed the preparation of numerous multilayered devices across multiple length scales by resorting to the LbL assembly technology. These comprise various objects at the nano‐ to macro‐scale, which can either be 2D or 3D.

### 2.1 2D LbL Assemblies

In the 2D category, also termed “surface coatings,” we find multilayered thin films,^[^ ^26^, ^27^ ^]^ which are deposited on a planar solid substrate, and micropatterned films, which are assembled on micro‐textured planar solid templates. In addition, the LbL technology also enables the buildup of enhanced cell signaling microenvironments on pre‐formed structures so as to instruct and guide cell fate both in vitro and in vivo.^[^ ^28^ ^]^

Multilayered films can be easily detached from an underlying low surface energy template, without any chemical or physical stimuli, in order to form robust and thicker free‐standing multilayered membranes, whose topography recreates the template in which the multilayered films have been assembled.^[^ ^29^, ^30^, ^31^, ^32^ ^]^

The complex composition, internal architecture, and highly aligned multiscale organization of tissue, such as muscle, nerves, and bone, have inspired the design and development of complex and innovative 2D multilayered nano‐ and micro‐devices capable of better recreating the features of their native cellular microenvironments so as to ensure better tissue‐biomaterial integration and performance in vivo, possibly facilitating their clinical translation.^[^ ^32^ ^]^

**Figure 2:**
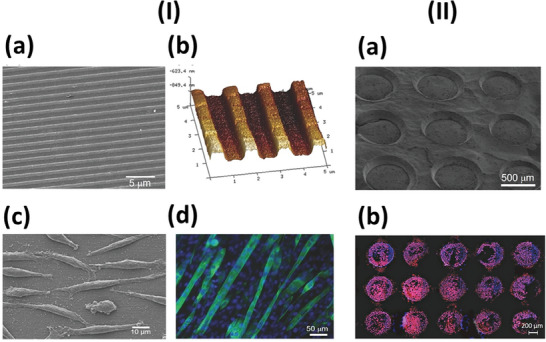
Patterned free‐standing multilayered membranes. I) Representative a) SEM and b) AFM images of polycarbonate template nanopatterned (CHT/CS)_300_ free‐standing multilayered membranes. Representative c) SEM micrograph of L929 cells seeded at 7 days of culture. d) Immunofluorescence image with troponin T (green myotubes) and DAPI (blue nuclei) staining of C2C12 myoblast cells at 10 days of culture. A differentiation medium was used, on polycarbonate template nanopatterned (CHT/CS)_300_ free‐standing multilayered membranes, revealing myoblast differentiation into mature myotubes. Reproduced with permission.^[^ ^32^ ^]^ Copyright 2017, WILEY‐VCH Verlag GmbH & Co. KGaA, Weinheim. II) Representative a) SEM and b) DAPI‐phalloidin fluorescence microscopy images of SaOs‐2 human osteoblast‐like cells at 7 days of culture on polydimethylsiloxane template micro‐patterned (CHT/ALG)_100_ free‐standing multilayered membranes. Reprinted with permission.^[^ ^33^ ^]^ Copyright 2017, Elsevier. ALG: alginate, CHT: chitosan, CS: chondroitin sulfate, SEM: scanning electron microscopy, AFM: atomic force microscopy.

In this regard, the fabrication of 2D patterned and easily detachable multilayered devices, denoting a well‐defined and precisely controlled nanopatterned design across the *XY* surface plane, has been in the spotlight. These multilayered devices are defined by the topography of the template and a multicomponent nanostructured organization, assigned by individual layers, along the vertical direction (*Z* axis). Sousa et al. reported the preparation of polycarbonate‐templated natural origin (chitosan (CHI)/chondroitin sulfate)_300_ (where 300 is the number of polymer bilayers) nano‐ and micropatterned cross‐linked free‐standing multilayered membranes denoting well‐defined extracellular matrix (ECM)‐mimetic nano‐grooved topographical features (**Figure** **2**I). Such membranes have promoted the adhesion, proliferation, migration, and alignment of C2C12 cells along the nanopattern direction, as well as their myogenic differentiation into myotubes,^[^ ^32^ ^]^ holding great promise for muscle tissue regeneration. Such an approach can be easily transposed to other cell types responding to topographical features, including neuronal cells, in order to trigger neuronal tissue regeneration, opening new avenues in regenerative medicine. Moreover, Martins et al. produced polydimethylsiloxane‐templated asymmetric (CHI/alginate (ALG))_100_ micropatterned self‐standing multilayered membranes denoting a well‐defined array of micropores in just one side, where osteoblast‐like cells tended to colonize preferentially (Figure 2II).^[^ ^33^ ^]^ These microwells can act as micro‐reservoirs, enabling the encapsulation, protection, and on‐demand targeted delivery of multiple bioactive agents, or even as cell carrier patches for multiple tissue engineering and regenerative medicine strategies. Among the multitude of devices that can be produced in an LbL fashion, 2D multilayered devices have indeed the greatest potential to be translated into the clinic,^[^ ^34^ ^]^ and be commercialized due to their easy and well‐established feasibility for automated large‐scale mass production.^[^ ^1^ ^]^

### 2.2 Advanced 3D Systems

Taking advantage of the high versatility offered by the LbL assembly technology, we have recently witnessed the LbL coating of more convoluted 3D surfaces, which have already paved their way in biomedicine, showing great promise in both in vitro and in vivo scenarios. These comprise paraffin wax‐coated glass‐templated hollow multilayered tubes,^[^ ^35^ ^]^ spherical paraffin wax‐templated moldable interconnected highly porous LbL macroconstructs,^[^ ^14^, ^36^ ^]^ spherical‐shaped core–shell multilayered nano/microparticles and hollow multilayered microcapsules,^[^ ^19^, ^37^, ^38^ ^]^ as well as liquefied ALG templated multilayered microcapsules.^[^ ^38^, ^39^ ^]^ In addition, the LbL films can be deposited at the surface of 3D scaffolds or be applied to coat dynamic living cells (see Section 2.3).

**Figure 3:**
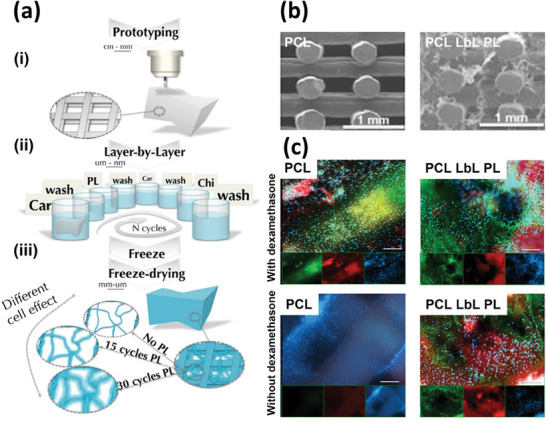
3D multiscale bioinstructive layer‐by‐layer (LbL) constructs. a) Schematic illustration of the macro/micro scaffolds prepared by rapid prototyping i) before and ii) after LbL coating with platelet lysate (PL) and multilayers of marine polysaccharides, and iii) freeze‐drying after the LbL assembly process. b) Representative SEM micrographs of freeze‐dried polycaprolactone (PCL) scaffolds before (left) and after LbL coating (right), showcasing the formation of nano/submicron fibrillar structures in the latter. c) Representative fluorescence microscopy images of human adipose‐derived stem cells (hASCs) after 4 days (basal media) plus 28 days of culture in osteogenic media (with dexamethasone) or osteoconductive (without dexamethasone). This microscopy image reveals the osteogenic differentiation of hASCs into osteoblasts in the case of the platelet lysate (PL)‐derived LbL‐coated PCL scaffolds, even in the absence of dexamethasone, although to a lesser extent than in the presence of dexamethasone. Stainings: human osteocalcin (green), Alizarin red (red), and cell nuclei (blue). The panels at the bottom showcase the images of each color channel. Scale bars: 200 µm. SEM: scanning electron microscopy. Adapted with permission.^[^ ^28^ ^]^ Copyright 2015, American Chemical Society.

In this regard, Oliveira et al. have coated prototypes of 3D poly‐caprolactone‐based porous macro‐scaffolds with cell‐instructive multilayer nanofilms. These nanofilms comprised alternate nanolayers of marine‐derived polysaccharides and human platelet lysates, so as to provide bio‐instructive cues and effectively apply them in bone tissue engineering (**Figure** **3**a). The developed 3D multiscale bio‐instructive constructs were shaped into hierarchical LbL sub‐micro/nano‐fibrillar structures in the inner environment by freeze‐drying (Figure 3b).^[^ ^28^ ^]^ These nanofibrillar structures act as enhanced cell‐anchorage points to promote the osteogenic differentiation of human adipose‐derived stem cells into mature osteoblasts in comparison with bare 3D scaffolds (Figure 3c). A wide array of tissue engineering strategies could be pursued, considering the customizable 3D scaffold design and inner ECM‐mimetic fibrillar architecture imparted by the LbL nanocoatings enclosing human platelet lysates as a pool of multiple proteins, growth factors, and other bioactive molecules.

It is possible to design and build 3D (multi)compartmentalized systems,^[^ ^40^, ^41^ ^]^ with a hierarchical organization from the nano‐ to the macroscale, reminiscent of living systems. The compartmentalized systems enable the efficient and simultaneous spatial confinement of a wide array of functional building blocks, including particles, capsules, bioactive agents, and living cells within a well‐controlled internal environment, holding great potential as smart carrier vehicles, customizable bioreactors, intracellular trafficking devices, bio‐mineralizable matrices, and as platforms for developing microtissues.

Therefore, the possibilities are numerous since numerous templates can be selected and coated with an unprecedented choice of biologically derived constituents using a wide range of assembly methodologies.

In view of their versatility, ease of preparation, and compatibility with “mild conditions” (e.g., physiological temperature, possibility to use water with salt as suspending medium), such multilayered devices hold great promise in the biomedical field, notably as carrier vehicles for the encapsulation and sustained release of model hydrophilic/hydrophobic bioactive agents,^[^ ^23^ ^]^ and as ECM mimetics to regulate cell functions,^[^ ^42^ ^]^ to name just a few examples.

### 2.3 LbL Coating on Cell Surfaces

Within the body, nearly all cells from any tissue reside in the fibrous nano‐meshwork of the native ECM, which regulates cell functions, including adhesion, growth, proliferation, differentiation, protein secretion, and 3D association.^[^ ^43^ ^]^ LbL assembly is a versatile and suitable method to construct highly customized ECM‐mimetic protein/protein or polymer/protein multilayers on different surfaces, including living cells, on account of its mild processing conditions.^[^ ^44^ ^]^ Besides the ECM proteins, cell membranes can be functionalized with virtually any desired biological component, including DNA, polysaccharides, or a combination thereof, enabling the assembly of cyto‐compatible cell multilayer shells coated with a nanosized ECM‐mimetic multicomponent composition, and thus multi‐functionalities, so as to address a broad range of applications in the biomedical field.

The LbL assembly technology simply relies on the alternating adsorption of interactive molecular solutions comprising complementary molecules without expensive or specialized instrumentations, complicated operations, or harsh environmental conditions. As a consequence, the mild LbL coating of living cell surfaces has attracted much interest in recapitulating the ECM microenvironment in order to better control cell functions and engineer hierarchically ordered 3D cell‐rich structures and artificial living tissues.

In this section, we summarize recent works on the LbL coating of cell surfaces, as well as their bio‐applications.

#### 2.3.1 Passive and Active Cell Surface Coatings by LbL Assembly

The LbL coating on red blood cells (RBCs) was reported for the first time in 2001.^[^ ^45^ ^]^ Bäumler et al. constructed poly(allylamine hydrochloride) (PAH) and poly(styrene sulfonate) (PSS) multilayers on the surface of fixed human RBCs as a template to prepare hollow microcapsules. This is a typical example of a simple fabrication of polyelectrolyte multilayer (PEM) films on cell surfaces without additional bioactivity. More recently, cell surface functionalization by LbL coating has received great interest as a tool enabling the *post*‐LbL addition of multiple complementary functional molecules at living cells'surfaces.^[^ ^46^ ^]^ In this section, we refer to the first approach as “passive coating” and the second as “active coating,” and summarize recent developments regarding these methods.

#### 2.3.2 Passive Coating of Cell Surface

**Figure 4:**
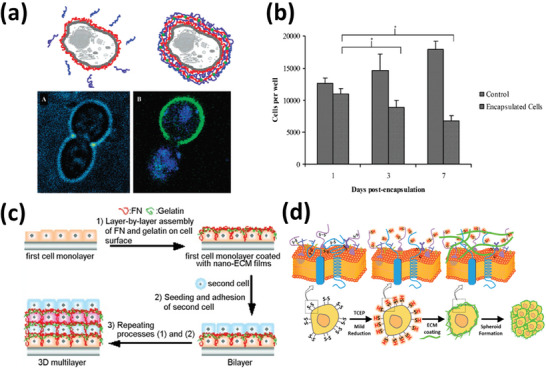
a) Upper: alternative adsorption of PAH polycation (red) and PSS polyanion (blue) on a single living cell. Green represents FITC‐labeled PAH. Lower: A) transmission image of a duplicating cell.^[^ ^48^ ^]^ Adapted with permission.^[^ ^48^ ^]^ Copyright 2002, American Chemical Society. b) Cell proliferation after coating with polyelectrolyte multilayers. Reproduced with permission.^[^ ^54^ ^]^ Copyright 2007, WILEY‐VCH Verlag GmbH & Co. KGaA, Weinheim. c) Fabrication process of 3D cell multilayers by depositing cells and fibronectin (FN)/gelatin nanofilms. Reproduced with permission.^[^ ^60^ ^]^ Copyright 2007, WILEY‐VCH Verlag GmbH & Co. KGaA, Weinheim. d) Schematic illustration of cell membrane modification and the coating of EMC proteins.^[^ ^69^ ^]^ ECM: extracellular matrix, FITC: fluorescein isothiocyanate, PAH: poly(allylamine hydrochloride), PSS: poly(styrene sulfonate), TCEP: tris(2‐carboxyethyl)phosphine. Adapted with permission.^[^ ^69^ ^]^ Copyright 2020, American Chemical Society.

Encapsulation of cells with appropriate materials offers a versatile approach to modulate communication within the cellular environment, minimize the host's hostile response to transplanted cells, and provide cell protection against physical damage.^[^ ^45^ ^]^ Following the initial investigation of PEM multilayer coating applied to fixed RBCs for the fabrication of hollow capsules, there has been a significant surge of interest in the encapsulation of living cells with PEM coatings. Lvov et al. successfully coated platelet surfaces with poly(dimethyldiallylammonium chloride) and PSS.^[^ ^47^ ^]^ Their results demonstrated that platelet aggregation and molecule secretion could be adjusted based on the thickness and components of the nanoshell. During the same year, Diaspro et al. studied cell encapsulation on yeasts by PAH/PSS multilayers.^[^ ^48^ ^]^ The authors found that the metabolic activities of encapsulated yeasts were preserved, given that they were still able to divide (**Figure** **4**a).

After achieving LbL encapsulation of living cells, several researchers concentrated on the biofunctions of coated cells. Li et al. observed the activation of the coated platelets by polyelectrolytes, such as poly(l‐lysine) (PLL) and PSS. However, no obvious activation was observed in platelets coated with glycol‐CHI and poly(l‐glutamic acid) (PGA) multilayers, thereby avoiding the release of growth factors and cytokines while maintaining platelet bio‐functions over a long time.^[^ ^49^ ^]^ Meanwhile, the nonimmunogenic PEM coating on RBCs not only maintained the cell viability and functionality, but also induced the immune‐camouflage of blood group antigens, facilitating the development of universal RBC production for medical transfusion.^[^ ^50^ ^]^ These results indicate that assembling multilayer films on cell surfaces using LbL technology not only maintains both cell viability and cell ability for growth and proliferation, but also alters the cell surface's properties which, in turn, affect the cell's response to its external environment. By varying either the assembly sequence or its components, the physicochemical properties of the nano‐shells can be effectively manipulated, thereby altering, or even enhancing the innate cell functions.

Following the successful coating of hemocytes and yeasts using the LbL assembly technology, the research focus has expanded to encompass the LbL encapsulation of a diverse range of mammalian cells, encompassing human cell lines. Chaikof et al. carried out many pioneering works on the encapsulation of pancreatic islets.^[^ ^51^ ^]^ These scientists achieved multilayer film formation on pancreatic islets using PLL‐*g*‐poly(ethylene glycol)‐biotin and streptavidin to mitigate deleterious host responses to transplantation.^[^ ^52^, ^53^ ^]^ The coated islets performed comparably to untreated controls in vivo in a murine model of allogenic intraportal islet transplantation. A similar coating possibility was shown using PEM coating on stem cells to improve cell viability during cell transplantation by Mills et al., who studied the encapsulation of mouse mesenchymal stem cells (MSCs) by PLL/hyaluronic acid (HA) multilayer films.^[^ ^54^ ^]^ A nanometric coating with a thickness of around 6–9 nm was obtained after the deposition of 10 PLL/HA layers. Although a significant decrease in cell number was detected after a couple of days in culture, which may be accounted for by the lack of film molecular permeability or PLL‐induced cytotoxicity (Figure 4b), LbL technology has made it possible to adjust the components or thickness of PEM shell to optimize its permeability. LbL PEM coating prolongs islet and MSC survival, and it is expected to be used for immune‐isolated transplantation in cell‐based therapeutic procedures. In addition to yeasts, beneficial microorganisms nanoencapsulated by LbL technology to maintain their viability, extend their shelf‐life, and control their release have also attracted increasing attention from medical, agricultural, industrial, and many other^[^ ^55^, ^56^ ^]^ fields. Altogether, the LbL technology offers a unique approach to reproduce the biochemical landscape of living cells. Due to the tunability of their composition and deposition frequency, the surface properties and permeability of the multilayer coatings can be tuned with nanometer precision so as to ensure the transport and release of signaling factors, oxygen, and other molecules, thereby laying the groundwork for a variety of biological applications.

#### 2.3.3 Active Coating of Cell Surfaces to Form Biomimetic Tissues

The incorporation of drugs, growth factors, antibodies, and other proteins in polyelectrolyte cell coatings is likely to offer new, interesting ways to influence cellular behavior. Depending on the requirements, using appropriate materials could promote cell proliferation, differentiation, achieve immune isolation, and prolong their circulation time in vivo. Therefore, LbL nano‐encapsulation of living cells has had a significant impact on many clinical areas including cell therapy, tissue engineering, and targeted^[^ ^57^, ^58^ ^]^ delivery. Lvov et al. deposited for the first time bovine immunoglobulins (IgG) on platelets, demonstrating that specific immune recognition and targeting with fluorescent anti‐IgG‐FITC, guide the targeted delivery of platelets in blood vessel through antibody coating.^[^ ^47^ ^]^ More recently, gold nanoparticles have even been introduced in the PEM coating on living cells, rendering it possible to remotely influence cells with light.^[^ ^59^ ^]^

Living cells can be coated in an LbL fashion so as to form 3D tissue‐like architectures,^[^ ^60^, ^61^ ^]^ with is a kind of “biomimetic tissue” made by the assembly of individual cells using engineering tools. An example is the cell coating using nanofilms made of the ECM proteins fibronectin (FN) and gelatin (G; denatured collagen) under mild conditions. Akashi et al. reported for the first time the cyto‐compatible LbL nano‐coating on living cell surfaces using FN and G (Figure 4c).^[^ ^60^ ^]^ FN/G nanofilms were specifically assembled on the cell membrane, although both molecules exhibit negative charges at physiological pH. FN can interact with G through a gelatin (collagen)‐binding domain in an FN subunit via biologically specific interactions. FN can likewise interact with cell membrane receptors, notably α5β1 integrins.^[^ ^62^ ^]^ Such nanofilms offer enhanced structural properties and functionalities to the surface‐engineered cells, thereby widening the array of applications. In particular, the biological‐specific interactions imparted by the FN/G nanofilms induce strong cell–cell interactions, thus enabling the fast bottom‐up assembly of a wide variety of artificial 3D in vitro tissue models and advancing cell‐based therapies.^[^ ^60^, ^61^, ^63^, ^64^, ^65^ ^]^ Apart from FN/G nanofilms, other protein/protein or even protein/polymer combinations have been explored for the functionalization of cell surface membranes to modulate cell functions and develop artificial 3D human in vitro tissue models.^[^ ^62^, ^66^, ^67^, ^68^ ^]^ Kim et al. recently reported a mild reduction of disulfide bond on human induced pluripotent stem cell (iPSC)‐derived MSC (iMSCs) surfaces using tris(2‐carboxyethyl)phosphine, and subsequent encapsulation with ECM.^[^ ^69^ ^]^ This mild reduction enhanced the coating efficiency without decreasing the cell viability and differentiation potential of iMSCs (Figure 4d). Similarly, to improve the efficiency of the cell surface coating, Hwang et al. demonstrated the mild disulfide bond reduction on cell surface for maleimide‐thiol conjugation.^[^ ^70^ ^]^ The reduced cells were successfully coated with biomolecules, polymers, and nanoparticles, with good viability preserved. However, in the case of PEMs, cationic polymers often induced strong cytotoxicity due to unspecific binding and cell membrane collapsing. Although various polyelectrolyte‐driven LbL nano‐films were tested, all nanofilms displayed cytotoxicity, with an intensity depending on their thickness, charge density, and polymer concentration.^[^ ^71^ ^]^ Accordingly, negatively charged or nonionic polymers are essential for coating the surface of living mammalian cells without cytotoxicity.

Overall, nanoencapsulation of living cells via LbL technology has proven to be an effective method to increase the survival and efficacy of cell transplantation. Meanwhile, nanometer‐scale artificial ECM films created using LbL technique, mimic the native ECM environment so as to promote cell adhesion and differentiation, facilitating the construction of 3D tissue models in vitro. Of note, the interactions between cells and their surrounding environment in vivo are extremely complex and variable. When constructing nano‐coatings in vitro, attention must be paid to these variations in order to meet cell/tissue culture's special needs at different stages. On the other hand, if we consider cells as building blocks, bioactive molecules and anti‐inflammatory drug delivery could be achieved using nano‐films as protective carriers^[^ ^23^ ^]^ Although the LbL cell coating technique has not yet been applied clinically, its simplicity, speed, and biological safety turn it into a feasible technology that could be applied to clinical settings in due course, subject to the compliance with regulatory issues and commercial scalability. As highlighted in the following section, LbL films have been developed for different applications both in vitro *and* in vivo.

## 3 LbL to Study Cell Signaling

### 3.1 Cell Response Modulation by Biophysical Cues

Biophysical cues (e.g., topographical features, stiffness) of the ECM play an essential role in modulating a large range of cellular processes, including cell adhesion, proliferation, differentiation, and migration.^[^ ^72^ ^]^ In this section, we discuss the relevance of the biophysical properties offered by PEMs for cardiovascular therapies.

Stiffness is an essential mechanical factor of ECM, which remarkably impacts cellular behavior. Over the last decades, several methods based on LbL have been developed in order to control surface stiffness so as to modulate cell behavior. Ji et al. introduced an LbL‐based strategy for modulating substrate stiffness just by regulating the thickness of PEMs coordinated with a rigid underlying substrate. This study demonstrated that increased LbL film thickness could decrease the surface stiffness sensed by the cells. By following this strategy, competitive adhesion of endothelial cells (ECs) over smooth muscle cells (SMCs) was achieved, given that the SMCs were more sensitive than ECs to decreased PEM stiffness.^[^ ^73^ ^]^ One of the most representative examples are films made of PLL and HA, whose stiffness can be regulated with adding 1‐ethyl‐3‐(3‐dimethylaminopropyl)carbodiimide (EDC) along with *N*‐hydroxysulfosuccinimide (sulfo‐NHS).^[^ ^74^ ^]^ LbL assemblies made of PLL and HA multilayers forming thin PLL/HA films (≈1.5 µm) can present with a controlled stiffness by modulating the concentration of EDC used to crosslink the films.^[^ ^75^ ^]^ By using the PLL/HA PEMs, Ji et al. systematically investigated the influence of surface stiffness of the endothelial‐to‐mesenchymal transition of ECs.^[^ ^76^ ^]^ They found that, though ECs prefer to adhere to a stiffer film, they lose their original functions, indicating that a soft substrate would be beneficial for preserving the original EC phenotype.^[^ ^76^ ^]^ Based on these findings, these authors combined PEMs with lower stiffness in combination with either soluble or matrix‐bound growth factors so as to promote the initial adhesion of ECs, thereby preserving the normal functions of the regenerated EC monolayer.^[^ ^77^, ^78^ ^]^

**Figure 5:**
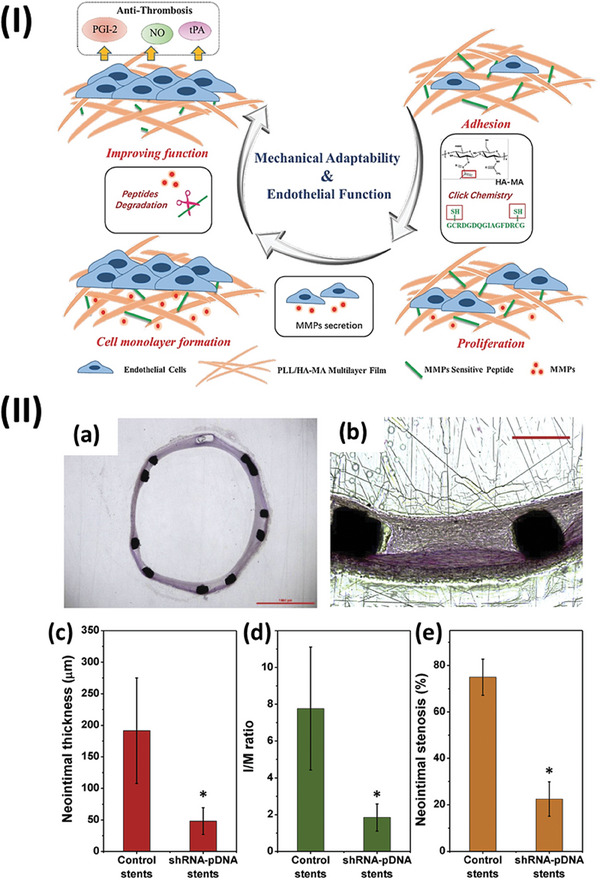
I) Schematic illustration of beneficial endothelial cell behaviors on stiffness‐adaptative (PLL/HA‐metacrylate) PEM crosslinked by matrix metalloproteinase sensitive peptides. Reproduced with permission.^[^ ^79^ ^]^ Copyright 2017, WILEY‐VCH Verlag GmbH & Co. KGaA, Weinheim. II) a,b) Typical optical photographs of the H&E stained cross‐section slices of the rabbit femoral arteries with shRNA‐pDNA stents. The scale bar of (a) represents 1 mm. The scale bar of (b) represents 200 mm. c) Histological analysis of neointimal thickness, d) I/M ratio, and e) percentage of neointimal stenosis (*p < 0.05).^[^ ^148^ ^]^ HA: hyaluronic acid, HA‐MA: methacrylated hyaluronic acid, I/M: intima/media, MMP: matrix metalloproteinase, NO: nitric oxide, PGI‐2: prostaglandin I2, PEM: polyelectrolyte multilayer, PLL: poly(l‐lysine), tPA: tissue plasminogen activator. Reprinted with permission.^[^ ^148^ ^]^ Copyright 2017, Elsevier.

As ECs are more likely to adhere to stiffer films while maintaining their healthy phenotype on softer films, the dynamic modulation of substrate stiffness can be used to improve EC. Given this context, Ji et al. introduced stiffness‐adaptive PEMs, fabricated using PLL and methacrylated HA (HA‐MA) and further crosslinked with matrix metalloproteinase (MMP)‐sensitive peptides.^[^ ^79^ ^]^ Their results demonstrated that EC adhesion was promoted by initial stiffer PEMs. The subsequent EC morphology and functions were improved via decreasing PEMs stiffness, as induced by the degradation of MMPs secreted by ECs (**Figure** **5**).^[^ ^79^ ^]^ The involvement of different stimuli‐responsive crosslinked bonds in PEMs likely renders the stiffness of PEMs changeable, after being exposed to different external stimuli. For example, due to oxidation and reduction reaction of disulfide bonds, the PEM made of PLL and thiol‐modified HA (HA‐SH) can be crosslinked by grafting thiol moieties to form PLL/HA‐SH multilayers. Such films can be crosslinked by adding chloramine T and further de‐crosslinked by the addition of glutathione.^[^ ^80^ ^]^ Moreover, the addition of azobenzene moieties could confer reversible crosslinking property to PEMs.^[^ ^81^ ^]^ These strategies offer numerous possibilities for stiffness modulation in various cardiovascular applications.

The topography of PEMs is another essential biophysical cue to regulate cell behavior. Constructing PEM on a patterned substrate is the most direct way to obtain a patterned PEM.^[^ ^82^ ^]^ The roughness of PEMs can be manipulated by controlling the fabrication conditions, including pH, ionic strength, molecular weight, and so on. Möhwald et al. found that salt concentration during LbL assembly greatly affected the rigidity and roughness of reducible hyperbranched poly(amidoamine)/DNA PEMs, rendering it easy to control various cell responses including viability, adhesion, transfection activity, and stress‐fiber orientation.^[^ ^83^ ^]^ In addition, McKee et al. employed PAA/PAH multilayers as soft lithography model, and found that the rough surface could downregulate the expression of several cardiovascular disease‐related EC genes.^[^ ^84^ ^]^

Another example of PEMs with topographical cues is the nature‐inspired ECM coating developed by Han et al., prepared by using a co‐culture of EC and SMC on HA and subsequent decellularization.^[^ ^85^ ^]^ This resulted in micro‐patterns, which improved the material's hemocompatibility, cytocompatibility, and tissue compatibility.

To enhance the differentiation of cells that are coated with LbL films, growth factors or functional polymers were employed as an intrinsic component of LbL multilayers. For example, vascular endothelial growth factor (VEGF) encapsulated into LbL films induced pre‐angiogenesis of human umbilical vein endothelial cells.^[^ ^86^ ^]^ Hong et al. demonstrated that collagen and alginic acid nanofilms fabricated on MSC surfaces promoted osteogenic differentiation, versus non‐coated cells.^[^ ^87^ ^]^ The FN/G nanofilms enhanced cell viability and beating strength of iPSC‐derived cardiomyocytes so as to produce injectable beating mini‐heart tissues via microfluidics. Basement membrane‐like LbL multilayers were reported using Type IV collagen and laminin to inhibit cell migration in 3D tissues.^[^ ^46^ ^]^ Tannic acid (TA) is another component that has been employed to generate functional LbL nanofilms. Choi et al. reported Fe^3+^‐TA nanofilms prepared on yeast cell surface to achieve a rapid degradation of the coating.^[^ ^88^ ^]^ The films were rapidly degraded under mild conditions via ascorbic acid‐mediated Fe^3+^ reduction, which allowed for overcoming the problems of metal‐organic complex disassembly, such as slow reaction rates and reaction incompatibility with living cells. They also reported hydrolysate and TA LbL films for protection and post‐functionalization of yeast cells.^[^ ^89^ ^]^

LbL assemblies are promising candidates to mimic the in vivo extracellular microenvironment, with precise control over the properties of the film architecture (e.g., thickness, stiffness). This method also allows for presenting biomolecules, such as peptides and proteins to cells.^[^ ^90^ ^]^

LbL films can be employed to deliver growth factors to trigger specific physiological processes.^[^ ^23^ ^]^ They can also be used to investigate cellular behavior and downstream signaling induced by these growth factors, such as bone morphogenetic proteins (BMPs),^[^ ^91^, ^92^, ^93^, ^94^ ^]^ and the stromal‐derived factor one (SDF1α).^[^ ^95^, ^96^ ^]^

### 3.2 Signaling by Bioactive Molecules, Growth Factors, and Peptides

Bioactive molecules can be presented at the surface of the LbL films or embedded into it either via incorporation during film buildup or via passive physical adsorption after film buildup.^[^ ^21^ ^]^

An example is the PLL/HA films, which can trap growth factors by physical adsorption, that is, without covalent linkage.^[^ ^97^ ^]^ The growth factors are presented to cells in a matrix‐bound manner, referred to as bBMP‐2.^[^ ^97^ ^]^

#### 3.2.1 BMP Signaling

BMPs are potent osteo‐inductive proteins involved in numerous physiological and pathological processes, including cancer development.^[^ ^98^ ^]^ In this section, we will review how growth factors like BMPs can be presented to cells via the LbL films for two major applications, namely to conduct fundamental studies on cell signaling, and to deliver BMPs at the implant site to trigger localized bone regeneration.

(PLL/HA)*_n_* biomimetic films were used to assess the effect of BMP‐mediated signaling, which is usually followed by quantifying the phosphorylation level of SMAD transcription factor. In the initial studies, the delivery of soluble (sBMP‐2) or bBMP‐2 was assessed in combination with either soft or stiff (PLL/HA)*_n_* films (stiffness was controlled from a few tens of kPa until several hundreds of kPa by adjusting the level of film crosslinking). SMAD phosphorylation was higher in the conditions of bBMP‐2 on soft films, in comparison with sBMP‐2.^[^ ^92^ ^]^ Unexpectedly, besides BMP‐2‐mediated signaling, bBMP‐2 was able to induce cell adhesion and spreading on soft films that are normally poorly adhesive for cells. Moreover, cell migration was potentiated on both soft and stiff films when BMP‐2 was present in the films. This BMP‐2‐induced cell spreading was mostly mediated by the adhesion receptor of the integrin family, named β3 integrins, and by a Type I BMP receptor, named ALK3 (BMPR1A).^[^ ^91^ ^]^ BMP receptors and β3 integrin were found to work together to control both SMAD signaling and cell adhesion.

The fact that bBMP‐2 is not covalently bound to the film enables it to study BMP‐2 internalization, and the effect of film stiffness on BMP‐2 internalization.^[^ ^99^ ^]^ Interestingly, BMP‐2 internalization was strongly stiffness‐dependent, given that the internalization was five times faster when BMP‐2 was internalized from a soft versus stiff film, with the amount of internalized BMP‐2 being increased by eightfold. The presentation mode (sBMP‐2 versus bBMP‐2) did not influence BMP‐2 internalization for soft films. However, considering stiff films, the internalization was much lower and slower for bBMP‐2.

Very recently, the BMP2‐presenting films were used to study the role of the BMP receptor ALK3 in BMP‐mediated cell signaling.^[^ ^100^ ^]^

Interestingly, to spatially control cell differentiation, BMP‐2 can be printed onto the LbL films, where it remains bioactive.^[^ ^101^ ^]^ For instance, single‐cell micropatterns of FN/BMP‐2 on soft films were used to study the mechanisms of BMP‐2‐mediated mechano‐transduction.^[^ ^102^ ^]^ Cell spreading itself potentiated the phosphorylation of SMAD, which depended on BMP‐2. This result differed from the effect of MSC spreading when cultured on stiff substrates in the presence of sBMP‐2. In this case, cell differentiation was found to be regulated solely by another pathway involving the RhoA kinase.^[^ ^103^ ^]^

#### 3.2.2 SDF‐1α Signaling

**Figure 6:**
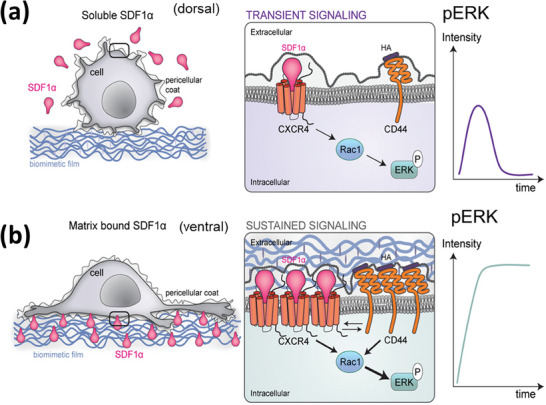
Schematic illustration summarizing the major differences in MDA‐MB‐231 cancer cell response to SDF‐1α presented either a) as soluble cue or b) as matrix‐bound cue for MDA‐MB‐231 breast cancer cells at different length scales from left to right. Left: at the cellular scale; middle: at the plasma membrane/receptor scale, and right: consequence in terms of phospho‐ERK signaling. a) In the case of a soluble presentation (sSDF), the local SDF‐1α concentration is low and SDF‐1α is mostly presented at the dorsal side of the cells. As a consequence, cell adhesion is low and cells are round with only a few protrusions. SDF‐1α‐induced signaling via CXCR4 activates Rac1 and pERK, but the intensity of the signal is small and its duration is transient (right illustration). b) In the case of matrix‐bound SDF‐1α (bSDF), the local concentration of SDF‐1α in the polyelectrolyte film is locally very high. As a consequence, the cellular receptors (CXCR4 and CD44) localized at the cell membrane close to the LbL film can bind to their SDF‐1α ligands and hyaluronic acid. This induces CXCR4 and CD44 receptor clustering (middle illustration). In this situation of spatial confinement, the two receptors act in concert and activate Rac1 and the subsequent phosphorylation of ERK1/2. Therefore, the intensity of the pERK signal is very high and is sustained over a long time, for at least 16 h, as shown. Reproduced with permission.^[^ ^96^ ^]^ Copyright 2017, Elsevier. SDF1α: stromal cell‐derived factor‐1 alpha, HA: hyaluronic acid, ERK: extracellular signal‐regulated kinase, CXCR4: C‐X‐C chemokine receptor Type 4, Rac1: rac family small GTPase 1.

The chemokine SDF‐1α (also known as CXCL12) is another example of a potent bioactive chemoattractant, with a crucial role in hematopoietic stem cell homing^[^ ^104^ ^]^ and cancer progression.^[^ ^105^ ^]^ LbL films were used to present SDF‐1α in a matrix‐bound manner to assess its functions on myogenic differentiation.^[^ ^95^ ^]^ The matrix‐bound SDF‐1α (bSDF) was found able to enhance myogenic differentiation by promoting troponin T expression more efficiently than the soluble one, as a function of culture time. However, the myotube fusion process was impaired, resulting in small myotubes with a few nuclei. In another study, a biomimetic tumoral niche was obtained by using a thin and soft LbL film that presented the SDF1a in a matrix‐bound manner to breast cancer cells.^[^ ^96^ ^]^ Potent cellular effects could be revealed and previously hidden molecular mechanisms have been identified using this chemokine presentation mode^[^ ^96^ ^]^ (**Figure** **6**), which could not be identified with the soluble SDF1. SDF1 presented in a matrix‐bound manner induced a striking increase in cell spreading and migration, which was accompanied by protrusion formation (e.g., lamellipodia, filopodia) specifically mediated by CXCR4 in MDA‐MB231 cells. CD44, the major hyaluronan receptor, was further found to act in concert via a spatial coincidence. The CXCR4/CD44 mediated cellular response to bSDF was found to be dependent on the Rac1 RhoGTPase, which was maintained only in the bSDF condition. Conversely, the transient signaling occurred in response to soluble SDF‐1α.^[^ ^96^ ^]^

Another example is transforming growth factor (TGF)‐β1, a crucial member of the TGF family, which induces epithelial‐mesenchymal transition (EMT) in tumor progression and metastasis.^[^ ^106^ ^]^ A tumoral ECM‐mimicking matrix was made using LbL films with controllable stiffness, with dopamine used to immobilize TGF‐β1 with a high efficiency of ≈90%.^[^ ^107^ ^]^ The stiff matrix with immobilized TGF‐β1 (i‐TGF) was the only condition able to induce mesenchymal phenotype and promote the EMT progression of hepatocellular carcinoma cells. The TβRI receptor was found able to enhance mechano‐sensor β1 integrin expression, followed by a significant activation of FAK and PI3K phosphorylation.

Overall, these examples using growth factors show that their cellular effects can be potentiated when they are presented to cells via LbL films, in a matrix‐bound manner, the stiffness of the LbL films having likewise an impact on cellular processes.

#### 3.2.3 Bioactive Peptides

Peptides can be grafted to one of the polyelectrolytes constituting the LbL film and thus be used to target cellular receptors. Proof‐of‐concept of targeting the cellular receptors integrins, involved in cell adhesion, was performed using the RGD (Arg‐Gly‐Asp) tripeptide.^[^ ^108^ ^]^ RGD presentation from the soft films, as well as higher film stiffness, could both induce cell adhesion. However, the integrins involved in cell adhesion on soft and stiff films differed. Soft films presenting RGD peptide appeared to be appropriate to induce myogenic differentiation, whereas the stiff PLL/PGA films tended to induce higher cell migration and proliferation, while inhibiting myogenic differentiation. Thus, different signaling pathways may be activated by modulating mechanical and biochemical properties of multilayer films.

In another work, PEM films presenting two different peptides derived from skeletal muscle ECM proteins were employed to target syndecan and/or integrin receptors.^[^ ^109^ ^]^ Lamellipodia formation, increased migration speed, directionality, and cell proliferation were observed on laminin‐derived peptide‐presenting films targeting syndecan‐1, whereas myotube formation was impaired on such films. Further investigations revealed that this peptide acted on signaling pathways involving Rac1 and Cdc42 kinases and β1 integrin adhesion receptor. On the contrary, RGD‐containing peptide allowed for cell adhesion via β3 integrins and promoted muscle cell differentiation. These results demonstrated that peptides grafted onto multilayered films could influence the proliferation/differentiation balance, while a crosstalk between different adhesion receptors could be observed.

Recently, biocompatible and biomimetic PLL/HA LbL nanofilms functionalized with an outer positively‐charged layer encompassing the self‐assembling peptide amphiphile (PA) laminin‐mimetic pentapeptide IKVAV (Ile‐Lys‐Val‐Ala‐Val) were shown to promote neurite outgrowth.^[^ ^27^ ^]^ This nanofilm promoted the enhancement of primary neuronal cell adhesion, viability, and morphology versus the IKVAV‐free PA. This effect was due to the synergistic effect of the nanofibrillar surface morphology and neuronal cell‐instructive bioactive cues entailed by the self‐assembling IKVAV‐PA functionalized HA‐ended multilayered nanofilm. The negatively‐charged HA outer layer was shown to trigger the self‐assembling ability of the oppositely charged PA into nanofibers via charge neutralization. The high density of the IKVAV epitope adsorbed on the surface of the HA‐ended multilayered nanofilms was essential to enhance neuronal cell functions, stimulate neurite outgrowth, and trigger the formation of an advanced neuronal stage development. We envisage that such a minimalistic approach, combined with the simplicity and high versatility granted by LbL technology, could be applied to coat surfaces of virtually any size, geometry, and surface chemistry with any biomaterial combination aimed to elucidate cell‐biomaterial interactions and stimulate the desired cell‐signaling pathways in regenerative medicine strategies.

## 4 LbL Assemblies as Vaccine Delivery Systems

One of the main assets of the LbL technology relies on the possibility of incorporating a large variety of molecules into the assembly, ranging from synthetic polymers to proteins, polysaccharides, or lipids. This is of great interest for designing vaccine formulations where antigens could differ, ranging from a whole pathogen to either a recombinant protein or nucleic acid.^[^ ^110^ ^]^ A vaccine antigen is an entity that is likely to induce a specific immune response directed against a pathogen or tumor cells for cancer therapy. Once administered, the antigen will be carried in by antigen‐presenting cells (APCs) so as to initiate the adaptive immune response. Importantly, the vaccine administration route is paramount, as the induced response likely differs depending on the targeted compartment. Although most marketed vaccines are administered via intra‐muscular injection, alternative routes are also being explored, consisting of either parenteral (subcutaneous, intradermal) or mucosal (oral, nasal, buccal, or vaginal) delivery. Besides, in order to mimic virus‐like assembly, virus‐like particles or nanoparticulate antigen were developed. These vaccine antigens are potent immuno‐stimulators, which are administered in order to mimic natural infection by delivering the antigen by the same internalization process in APCs. Thus, depending on the antigen's nature, size, and stability, as well as their administration route, LbL delivery systems must be conceived accordingly.

Besides the acknowledged versatility offered by LbL assemblies, their main advantage lies in offering an efficient protection of antigens and adjuvants that they carry against enzymatic or physical degradation.^[^ ^111^ ^]^ Moreover, LbL assemblies allow for incorporating a controlled amount of cargo at a relatively high density.^[^ ^110^ ^]^ The following part will present several recent advances in the development of LbL‐based vaccine delivery systems.

### 4.1 Protein‐ or Peptide‐Based Vaccine Delivery

Protein subunit vaccines are composed of viral proteins or peptides (antigen) that are co‐delivered with a potent adjuvant in order to counterbalance their low immunogenicity compared with inactivated/attenuated vaccines. However, recent studies have highlighted the potential of LbL nanovectors to provide a significant adjuvant effect along with their antigen carrier function.^[^ ^112^ ^]^ Vaccine components can either be embedded or adsorbed on LbL to target APCs. In a recent study, an LbL mucoadhesive patch was developed for sublingual delivery of the p24 antigen (protein from human immunodeficiency virus) adsorbed on a PLA nanoparticle carrying an adjuvant.^[^ ^113^ ^]^ The LbL patch was composed of polysaccharides (chitosan and hyaluronic acid) and was used to increase the vaccine retention time in the sublingual mucosa.

Even though previous work has shown that LbL microcapsules, made of dextran sulfate and poly(l‐arginine), induced an adaptive immune response against a model protein antigen (ovalbumin, OVA) similar to that of poly(lactic‐*co*‐glycolic acid) (PLGA) microparticles, LbL assemblies benefit from the versatility triggered by the physico‐chemical properties of the polyelectrolyte‐pairs used.^[^ ^114^ ^]^ These polyelectrolytes can be chosen as structural building blocks or directly as immune stimulators.^[^ ^115^ ^]^ For example, the functionalization of mesoporous silica nanoparticles with LbL assembly of negatively‐charged lipid bilayers (DOPCDOPC, DOPS, and cholesterol) and positively charged *N*‐trimethyl chitosan was used for toxin (diphtheria toxoid) release from hollow microneedles.^[^ ^116^ ^]^ LbL nanoparticles of 400–600 nm were produced by assembly of PGA and PLL with a modified respiratory syncytial virus (RSV) G protein (peptide antigen) motif added as the external layer.^[^ ^117^ ^]^ These nanoparticles were shown to induce neutralizing antibodies against RSV G protein after subcutaneous injection in a mouse model. The produced antibodies were able to diminish the viral load after RSV infection and reduce inflammation in the respiratory tract. In this case, LbL technology was applied in order to increase the diameter of the particles from 150 nm for uncoated particles to 400–600 nm for LbL‐coated particles. Besides, the increase of local antigen concentration by adsorption of several peptides on a single particle is expected to improve the magnitude of the induced immune response. These particles were further optimized using an immunostimulant in order to specifically direct the immune response toward a more protective immunity against RSV infection.^[^ ^118^ ^]^

Micro‐needle platforms were thoroughly developed for intradermal vaccine administration through LbL assemblies of lipids or polymers.^[^ ^119^, ^120^ ^]^ LbL‐coated micro‐needles were used for delivering the protein antigen OVA with a sustained release over 3 days after a 1‐min skin injection to implant LbL films in vivo.^[^ ^120^ ^]^ Micro‐needle platform was also used for delivering a three‐component vaccine composed of the dengue virus envelope protein domain III (antigen) along with two adjuvants, consisting of the double‐stranded ribonucleic acid (dsRNA) poly(I:C) and an amphiphilic peptide.^[^ ^121^ ^]^ The degradation of the three‐component LbL film due to β‐aminoesters resulted in the formation of vaccine polyplexes with a sub‐micrometer size, and their release from the micro‐needle platform. One of the LbL technique's main advantages was the control of the three components’ release kinetics, with the absorbed peptide being the first to be released (clearance rate of 24 h), whereas the dsRNA and protein antigen remained at the injection site for 3 days and 2 weeks, respectively.

The control of temporal release and delivery sequence of vaccine components are key to developing efficient vaccine delivery systems, as they can modulate immune signaling and improve the adaptive immune response. Besides, LbL self‐assembly methods enabled the development of a self‐boosting vaccine, consisting of a one‐shot vaccine able to induce an antigen‐specific immune response similar to that of a prime‐boost vaccination scheme.^[^ ^122^ ^]^

Despite promising pre‐clinical advances, LbL vaccines are not yet recognized and approved for clinical use. Vaccines are highly controlled medicines, and more storage, dosage, and safety studies must be carried out before they can be approved.^[^ ^111^ ^]^

### 4.2 Nucleic Acid‐Based Vaccine Delivery

Although most of the development of LbL‐based vaccine delivery system focused on protein delivery or even whole virus incorporation,^[^ ^123^ ^]^ the LbL assembly likely offers great promise for the delivery of negatively‐charged nucleic acid‐based vaccines. Desoxyribonucleic acid (DNA) vaccines delivered by LbL nanocomplexes or LbL films allow for controlled or sequential DNA delivery by using biodegradable or non‐biodegradable polyelectrolytes.^[^ ^124^, ^125^ ^]^

DNA vaccines could be designed for preventive action (prophylactic) or therapeutic perspectives, including cancer vaccines. DNA‐based cancer vaccines rely on the modulation of the patient's immune system so as to identify and kill malignant cells by inducing antitumor immune responses. However, the development of such vaccines could be limited by their poor immunogenicity, partially due to their enzymatic degradation by DNases. Therefore, using LbL techniques to design effective anti‐tumor vaccines could be achieved by either protecting the cargo or targeting APCs aimed to improve the induced immune response without compromising on tolerance and safety. An efficient nucleic acid delivery system must induce a high transfection ratio, reflecting: i) the efficient uptake of the vaccine components by the targeted cell; ii) the effective escape of the nucleic acid from the endosome. This could be achieved by using pH‐sensitive polymers within the LbL assembly so as to favor DNA release in the acidic endosomal environment by LbL disassembly. Xu et al. reported the association of an amphiphilic lipopeptide (for APC targeting) and a charge‐reversible poly(allylamine hydrochloride)‐citraconic anhydride together with a mannosylated chitosan aimed to increase the release of the antigen‐encoded plasmid DNA.^[^ ^126^ ^]^ The ultra‐pH‐responsive polymer oligo sulfamethazine conjugated poly(β‐aminoester urethane) was also employed in combination with the poly(I:C) adjuvant to release vaccine polyplexes from polycarbonate microneedles after skin implantation.^[^ ^127^ ^]^

The recent COVID‐19 pandemic accelerated the emergence of mRNA‐based vaccines. Although the efficacy of liponanoparticle‐based formulations in protecting against severe disease forms was demonstrated, new delivery systems are currently being developed so as to decrease several possible adverse effects due to using PEGylated lipids,^[^ ^128^ ^]^ as well as to optimize formulations for mucosal administration.^[^ ^129^ ^]^ To this purpose, LbL‐based nanoparticles were developed to combine a solid PLA core and lipid/peptide layers containing mRNA molecules.^[^ ^130^ ^]^ The adsorption of mRNA molecules assembled with a cell‐penetrating peptide on the surface of a PLA nanoparticle covered by a lipid membrane enabled both cellular penetration and endosomal escape. As a perspective, these LbL hybrid lipid/polymer formulations could be more resistant to the shear stress experienced by nanoparticles during spraying, and they could thus be used for administration through the respiratory tract (nasal or pulmonary administration).

## 5 LbL Films for Tissue Engineering: Bone and Cardiovascular Tissue Engineering

### 5.1 Delivery of Growth Factors for Bone Tissue Engineering

In addition to these in vitro applications, LbL films can also be employed in vivo as carriers for growth factor delivery, in different fields of tissue engineering.

We herein present two examples in bone and cardiovascular tissue engineering and also show how LbL can be employed to engineer 3D tissues.

BMPs are the most widely used osteo‐inductive growth factors, being already employed in clinical practice. So far, only one material carrier has been approved by the United States Food and Drug Administration (FDA) for clinical use as a BMP carrier, which is the collagen sponge or paste. Collagen sponges loaded with BMP‐2 were FDA‐approved in 2002 as an alternative to bone graft. However, the low affinity between collagen and BMP‐2 requires using supra‐physiological BMP‐2 doses, which actually results in adverse effects, including ectopic bone formation.^[^ ^131^ ^]^ Moreover, this low affinity induces a burst of BMP‐2 release. Thus, the search continues for other carriers enabling the retention of BMP‐2 during a sufficient time (ideally 21 days) so as to mimic the physiological processes.^[^ ^132^ ^]^

LbL coatings are an interesting alternative to serve as a BMP‐2 reservoir. Indeed, these coatings can be designed to be biodegradable either via non‐specific hydrolysis of their components,^[^ ^133^ ^]^ or using a specific trigger.^[^ ^134^ ^]^ For example, Martin et al. synthetized a cytocompatible poly(thioketal *β*‐amino amide) polycation that is degradable by cell‐generated reactive oxygen species.^[^ ^134^ ^]^ These authors used this polycation in LbL coatings along with BMP‐2, which allowed for controlling its delivery upon oxidation. When implanted in vivo, the coatings that were the most sensitive to reactive oxygen species increased the extent of bone regeneration by 50% compared with coatings that were less sensitive to reactive oxygen species. Moreover, these coatings enabled a longer BMP‐2 delivery when compared to hydrolytically degraded LbL coatings. In another example, Chen et al. used an LbL coating of CHI and HA to load BMP‐2 and VEGF.^[^ ^135^ ^]^ In this study, BMP‐2 was post‐loaded into the coating.

It is possible to directly incorporate BMP‐2 as an LbL ingredient during the coating fabrication. Shah et al. employed this method and deposited tetralayers of polyelectrolytes containing a hydrolytically degradable polymer and BMP‐2.^[^ ^136^, ^137^ ^]^ The BMP‐2 amount was controlled by the number of deposits, and the degradability of the LbL film was controlled by synthetic polymer degradation. As an example, these authors used films made of 40 tetralayers of Poly2 (polycation), poly(acrylic acid) (polyanion), BMP‐2, and poly(acrylic acid).^[^ ^137^ ^]^ When 2 µg of BMP‐2 was loaded in the coating, ≈60% of BMP‐2 was released during the first day. This number was reduced to around 10% when 0.2 µg was loaded into the coating.

In another study, LbL films made of PLL/HA multilayers and loaded with BMP‐2 were deposited on polymeric tubes made of PLGA and implanted in a rat femoral bone defect.^[^ ^138^ ^]^ The presence of bone regeneration inside and around the tube with spatially distinct organization for trabecular‐like and cortical bones was demonstrated by means of histological staining and high‐resolution computed tomography. Interestingly, as the BMP‐2 dose increased, the cortical bone amount and thickness likewise increased.

**Figure 7:**
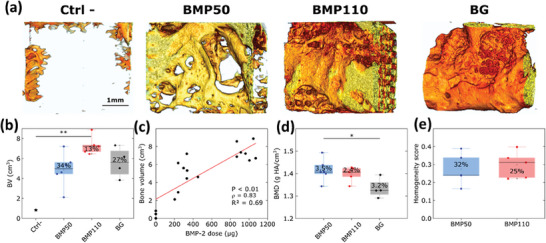
Quantitative CT analysis of bone formation at 3 months (D91) after explantation. a) Representative 3D reconstructions obtained for: the negative control (EDC30 film‐coated scaffold without BMP‐2), film‐coated scaffolds at BMP50 and BMP110, and bone autograft. b) Box plot representation of the total bone volume (BV) as a function of the experimental group. c) Total bone volume as a function of the total BMP‐2 dose per implant. d) Bone mineralized density (BMD) as a function of the experimental group. e) Homogeneity score measured for BMP50 and BMP110. The coefficient of variation is expressed as percentages in each box. *p < 0.05; **p < 0.01. Reproduced with permission.^[^ ^34^ ^]^ Copyright 2021, Elsevier. CT: computed tomography, BG: bone graft.

More recently, it was shown that such films combined with 3D polymeric scaffolds made by 3D printing of a synthetic polymer (polylactic acid) allowed for repairing a mandibular bone defect in mini‐pigs.^[^ ^34^ ^]^ The amount of regenerated bone and the repair kinetics were found to be significantly influenced by the BMP‐2 dose released from the scaffold (**Figure** **7**A). The bone was homogeneously formed inside the scaffold, and no ectopic bone formation was observed. The bone repair was as good as the bone autograft. Of note, the BMP‐2 doses applied in this study were 20 to 75 times lower than those of commercial collagen sponges used in current clinical applications. No adverse effects were observed. In addition to being efficient, such reduced doses provide a safer and cost‐effective way to deliver BMP‐2.^[^ ^139^ ^]^

### 5.2 Biomolecule Delivery for Cardiovascular Applications

Cardiovascular diseases are the leading cause of death worldwide, with about 17.9 million people dying every year, as reported by the World Health Organization. Over the last two decades, cardiovascular stent placement within a coronary artery to open narrowed or weakened arteries has been widely recognized as the gold standard for managing cardiovascular diseases.^[^ ^140^ ^]^ In order to prevent restenosis caused by bare metal stents, a drug‐eluting stent (DESs) carrying antiproliferative medication has been proven effective in slowing tissue overgrowth within the stent. However, clinical evidence revealed drug‐eluting stents to be likely to increase the risk for late stent thrombosis due to incomplete and delayed endothelium regeneration, which would be life‐threatening for patients.^[^ ^141^, ^142^ ^]^

In this context, it is ideal, although extremely challenging, to develop stents inducing better endothelium regeneration while inhibiting restenosis. The stent surface modification with functional PEMs via LbL assembly has shown great potential in promoting endothelization and preventing neointimal hyperplasia. PEMs can create a biomimetic microenvironment and modulate cell responses (e.g., adhesion, migration, proliferation, etc.) by enabling cells’ exposure to the desired bioactive agents (biochemical cues) and/or specific mechanical properties (biophysical cues).^[^ ^13^, ^24^ ^]^ In addition, recent developments in the preparation of PEM coatings using the industrial process render the scale‐up of the manufacturing of PEM‐coated stents in the future easy. In the following section, the cardiovascular applications of PEMs are reviewed.

As PEMs can be used as a delivery system of bioactive agents, they have attracted considerable interest in view of managing cardiovascular diseases.^[^ ^143^ ^]^ These bioactive agents (e.g., drugs, DNAs/RNAs, antibodies, peptides, growth factors, and enzymes) can be combined with stents and delivered to the desired site using stent deployment in order to further control cellular behavior locally.^[^ ^144^ ^]^ Various strategies have been introduced to load bioactive species inside PEMs, which are mainly classified into chemical modification and physical adsorption.

Electrostatic interaction being the main driving force behind PEM production, a large variety of charged biomacromolecules (e.g., peptides, antibodies, DNA, RNA, etc.) are directly used as PEM components.^[^ ^144^ ^]^ Lynn et al. constructed multilayer films on catheter balloons by alternately adsorbing layers of a hydrolytically degradable poly(β‐amino ester) and plasmid DNA, which successfully achieved local delivery of pDNA to vascular tissue.^[^ ^145^ ^]^ Indeed, pDNA can be encoded with different information so as to induce desirable functions. For instance, Ji et al. introduced a PEM‐mediated functional gene delivery consisting of protamine sulfate and plasmid DNA encoding hepatocyte growth factor (HGF‐pDNA). In the co‐culture system of EC and SMC, the PrS/HGF‐pDNA PEM transfected EC and SMC simultaneously, thus allowing them both to secrete HGF, which only promoted EC proliferation. Consequently, the competitiveness of EC over SMC was enhanced.^[^ ^146^ ^]^ Furthermore, these authors demonstrated that stents coated with PrS/HGF‐pDNA PEM could reduce in‐stent restenosis in vivo.^[^ ^147^ ^]^ Based on this study, Ji et al. developed another functional gene PEM using a pDNA encoding short hairpin RNA instead of HGF‐pDNA, which lowered the secretion of FN and collagen, inhibited the proliferation of fibroblasts, and downregulated the expression of TGF‐β1.^[^ ^148^ ^]^ In vivo, the arteries treated with the PEM‐coated stents showed significantly lower neointimal hyperplasia, neointimal thickness, intima‐to‐media ratio (I/M ratio), and neointimal stenosis rate when compared with bare stent‐treated arteries (Figure 7B).^[^ ^148^ ^]^ Despite functional genes, other biomacromolecules like heparin and collagen were employed to produce PEMs capable of inducing specific adhesion and growth of vascular ECs.^[^ ^149^ ^]^ It is worth mentioning that PEMs loaded with functional biomacromolecules regulate cell responses mainly in a surface‐mediated manner, differing from functional biomacromolecules acting in solution.

Since polyelectrolytes contain abundant functional groups, PEMs can be chemically modified using bioactive agents for cardiovascular purposes.^[^ ^150^ ^]^ Ji et al. immobilized anti‐CD34 antibody onto heparin/collagen PEMs through glutaraldehyde, resulting in a significantly decreased neointimal hyperplasia compared with unmodified heparin/collagen PEM.^[^ ^149^ ^]^ In addition, Mela et al. coated coronary stents with PEMs and subsequently introduced elastin‐like recombinamers (ELRs) inside the PEM via catalyst‐free click chemistry.^[^ ^151^ ^]^ The ELR‐covered stents exhibited great potential in inhibiting the formation of atherosclerotic plaque from the bloodstream, while promoting endothelization.^[^ ^151^ ^]^

Bioactive agents can be incorporated into PEMs via a “matrix‐bound” strategy, conferring specific biological functions to the PEMs.^[^ ^152^ ^]^ For instance, Ji et al. developed a PLL/HA PEM with matrix‐bound VEGF, thus demonstrating their ability to selectively promote EC adhesion and competitive growth.^[^ ^77^ ^]^ Recently, Ji's group focused on the development of functional PEMs as drug delivery systems. They introduced the polyethyleneimine/poly(acrylic acid) (PEI/PAA) PEM with a microporous structure by using acidic treatment and freeze‐drying. This porous PEM can be used as a drug reservoir for different biomedical purposes after absorbing the functional agents into the film by diffusion.^[^ ^153^, ^154^ ^]^ To demonstrate its potential in cardiovascular applications, the VEGF was encapsulated into the PEM, resulting in better adhesion of ECs on surfaces compared with the blank PEM in vitro.^[^ ^155^ ^]^

Although the LbL assembly method is a promising approach conferring desirable bioactive properties to cardiovascular implantable devices like vascular stents and balloons, challenges still remain regarding the translation from bench to bedside. First, the dynamic nature of bloodstream circulation within real blood vessels creates a complex environment.^[^ ^156^ ^]^ Nevertheless, most studies exploring the influence of biomolecule‐loaded PEM on endothelialization have been conducted in static settings. The shear stress induced by blood flow significantly impacts the stability and functional properties of the PEM. For example, the increase in flow rates results in a decreased ability of ECs to uptake updated nanoparticles.^[^ ^157^ ^]^ Therefore, whether PEM‐mediated functional gene delivery can successfully transfer ECs under flow shear stress is worth further investigating. Second, sterilization of cardiovascular implantable devices immobilized with biomolecules is another issue. The use of terminal sterilization methods, such as ethylene oxide, gamma radiation, and electron beams, can lead to activity loss within biomolecules.^[^ ^158^ ^]^ A previous study conducted by Ji's group revealed that PEM could protect loaded genes during industrial sterilization processes.^[^ ^147^ ^]^ However, further studies are still needed to further investigate this promising function of PEMs in improving the stability of loaded biomolecules. Furthermore, biodegradable cardiovascular implantable devices offer a promising alternative to non‐biodegradable ones.^[^ ^159^ ^]^ Most studies still use PEMs to modify traditional non‐biodegradable metal stents for in vivo experiments. To match the degradable period of the stents, developing PEMs with sustained release of biomolecules would produce long‐lasting therapeutic effects.

### 5.3 Biomedical Applications of 3D‐Engineered Tissues by LbL Cell Surface Coating

One application of cell surface coating by LbL nanofilms is to induce cell–cell interaction to build 3D cellular constructs, including spheroids or multilayers. These structures can be used for implantation in regenerative medicine or for drug assessment in drug discovery. Here, we summarize recent developments classified by targeted tissue type.

#### 5.3.1 Liver Tissues

Rajagopalan et al. reported on 3D liver models incorporating three different cell types (hepatocytes, liver sinusoidal endothelial cells, and Kupffer cells) using CHI/HA PEMs prepared on cell surfaces.^[^ ^160^ ^]^ The resulting 3D cell co‐cultures can be used as an organotypic hepatic model to promote proliferation and maintain phenotype as well as cell proportions in vivo. Akashi et al. used the “cell accumulation technique” by accumulating single cells encapsulated with nanometer‐sized ECM films of FN/G. FN/G nanofilms could stimulate cell–cell interactions to build vascularized 3D liver models.^[^ ^161^ ^]^ Since the coating induced higher cytochrome activity, it holds great potential for both regenerative medicine and drug discovery.

#### 5.3.2 Blood Vessels

Blood vessel wall‐like multilayered tissues were constructed using endothelial cells and SMCs by cell surface coating with FN/G nanofilms, which induced cell–cell interactions.^[^ ^162^ ^]^ Nitric oxide diffusion from endothelial cells into SMC multilayers was quantified in 3D using sensor particles. This 3D blood vessel wall model was shown useful to optimize nanoparticle size and cytocompatibility before animal experiments.^[^ ^163^ ^]^ In another work, Chan et al. studied the LbL seeding of SMCs in a microchannel scaffold with a thin collagen Type I hydrogel, building the 3D microarchitecture with aligned structures.^[^ ^164^ ^]^

#### 5.3.3 Heart Tissue

**Figure 8:**
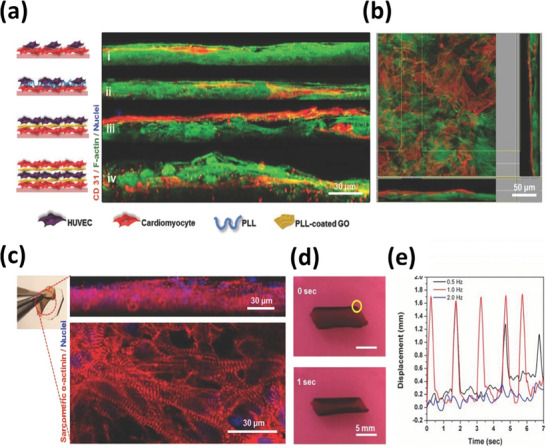
a) Schematic illustration (left) and 3D reconstructed confocal cross‐section images (right) of 2L cardiomyocytes and ECs without any ECM layers. Cardiomyocytes and ECs were stained with phalloidin (green), ECs were immunostained with CD31 (red), and nuclei were stained with DAPI (blue) after 5 days of culture. b) Confocal images (top view) of sample iii) showing widespread EC networks. c) A photograph of the peeling process of cellular construct and the confocal images of the interconnected sarcomeric structures (top image: cross‐section; bottom image: top view) of the 2L cardiomyocyte construct after 3 days of incubation. The cardiac cells were immunostained with sarcomeric α‐actinin (red) and nuclei with DAPI (blue). d) Optical images of the 2L cardiac actuator. e) Displacement of the actuator (focusing on the tip inside the d) yellow circle) over time under electrical stimulation (square wave, 1.4 V cm^−1^, frequency: 0.5–2 Hz, pulse width: 50 ms). Reproduced with permission.^[^ ^165^ ^]^ Copyright 2014, WILEY‐VCH Verlag GmbH & Co. KGaA, Weinheim. HUVEC: human umbilical vein endothelial cell, PLL: poly(l‐lysine), GO: graphene oxide, EC: endothelial cell, ECM: extracellular matrix.

The 3D‐cardiac tissue construction is most important in the drug discovery domain, as cardiac toxicity is still a significant concern. Graphene oxide‐gelatin methacrylate and PLL were employed as components of LbL coating on cardiac myocytes’ surfaces so as to construct 3D heart tissues, enabling assessing cardiac function and drug effects.^[^ ^165^ ^]^ By controlling the amount of graphene oxide used for the film construction, the thickness of the multilayer tissues could be tuned, while preserving good cell viability (**Figure** **8**). FN/G nanofilms were employed to produce a multilayered tissue construct of human iPSC‐derived cardiac myocytes, inducing cell‐ECM interaction. Akashi et al. performed a tensile test of the constructed iPSC‐derived cardiac myocyte tissues,^[^ ^166^ ^]^ and aligned tissue formation using dispensing instruments to enhance beating property.^[^ ^161^ ^]^

#### 5.3.4 Cancer Model

Human cancer model is one of the most desired 3D tissue models for toxicity and drug efficacy assessments, as monolayer culture does not reproduce the in vivo environment. Therefore, 3D culture systems are now expected to provide responses similar to the in vivo scenario. Moreover, human 3D cancer models are expected to better mimic human treatment reactions than animal experiments. Although the patient‐derived xenograft mouse^[^ ^167^ ^]^ is currently the only way to expand patient cancer cells without any gene modification, the high cost, low‐throughput, and low reproducibility are still major issues. Given this context, Chen et al. reported a mild method able to nanoencapsulate individual mammalian cells by LbL assembly using negative and positive gelatin inner layers and polyethylene glycol (PEG) outer layer crosslinking using succinimide thioether linkage. Nanoencapsulated individual HeLa cells displayed high survival rates and long survival periods, while being effective against large external entities and higher physical stresses. Controlled cell release is likely facilitated through the targeted cleavage of the external PEG layer.^[^ ^67^ ^]^ FN/G nanofilms are nano‐ECM that induce cell–cell interaction. By taking advantage of this property, cancer stromal tissues composed of fibroblasts and blood capillary‐like networks were constructed using the FN/G cell surface coating of pancreatic cancer cells culture at stromal tissues’ top surfaces.^[^ ^168^ ^]^ Cultured cancer cells strongly invaded the capillary blood network, similar to the primary metastasis process, rendering this cancer model potentially useful to assess the efficacy of anticancer drugs.

#### 5.3.5 Other Models

The other models, involving skeletal muscle,^[^ ^169^ ^]^ skin with vascular network,^[^ ^170^ ^]^ placental barrier models,^[^ ^171^ ^]^ and β‐cell spheroids,^[^ ^63^ ^]^ have been constructed using cell surfaces coated with FN/G nanofilms.

Given that FN and G are typical cell adhesive proteins interacting with the adhesion integrin receptors, encapsulated cells could immediately accumulate to a well‐structured and dense spheroid. The insulin secretion ability of the coated β‐cell spheroids was greater than that of uncoated ones. Moreover, cells with coating were also found to exhibit upregulated expression of connexin 36, a gap junction molecule. In vivo implantation experiments showed an immediate decrease in blood glucose levels and significant increase in glucose sensitivity after intraperitoneal glucose stimulation compared with uncoated spheres. These results suggested that the FN/G coating itself likely improves cell function.

In summary, these results demonstrate the relevance of cell environments on cell function in tissue engineering applications. In addition, they underline how versatile LbL cell coatings are and how they can improve tissue formation and function. Nanometer‐sized multilayers provide an ideal EMC‐mimic environment for cells, as well as an adhesive surface for a second cells type, which is likely critical for the successful application of these tissues in regenerative medicine. The precise manipulation of the cellular surfaces’ physicochemical properties at the nanoscale level actually facilitates the creation of a more accurate cellular environment that enables the customization of tissue properties. Particularly noteworthy is the application of the “cell accumulation technique,” which facilitates the easy adjustment of tissue thickness, as well as the composition and spatial distribution of various cell types, by modulating the number and sequence of seeding cells. This technique paves the way for the precise construction of complex tissue structures with different regions possessing distinct characteristics via bioprinting. In addition, nanometer‐scale ECM layers additionally protect cells against physical damage.^[^ ^172^ ^]^ Although LbL technology offers precise control of cells and cellular environment, challenges remain regarding the construction of tissues with clinically relevant sizes. Reproducibility is also a persistent issue, especially when considering its clinical application.

## 6 Recent Technological Developments

### 6.1 New LbL Coating Methods

The conventional method of LbL deposition is dip‐coating, which consists of alternately immersing virtually any solid substrate in complementary material solutions, while rinsing at each step to remove weakly adsorbed molecules. More recent developments include spraying, spin‐coating, brushing, and electromagnetic‐driven deposition, among others.^[^ ^173^ ^]^

Some of the techniques, such as spraying or electromagnetic‐driven deposition, aim at decreasing the time and amount of material needed for LbL film formation, while brushing enables the creation of patterned surfaces that can be used for tissue engineering strategies. Thus, Iqbal et al. designed hydrogen‐bonded TA/collagen LbL nanofilms to induce muscle fiber orientation during differentiation.^[^ ^174^ ^]^ Inkjet printing can also be used for LbL deposition as shown in 2010 by the team of Nicholas Kotov.^[^ ^175^ ^]^

A large overview of the numerous LbL assembly methods has been recently reviewed by Richardson et al.^[^ ^1^ ^]^

New approaches involve the combination of 3D printing with LbL deposition. In a recent work by Sousa et al.,^[^ ^176^ ^]^ the researchers employed 3D‐printed alginate sacrificial microstructures coated by CHI and arginine–glycine–aspartic acid‐couple ALG (ALG‐RGD). Shear‐thinning photocrosslinkable xanthan gum hydrogels exposed to a calcium‐chelating solution were used for the construct. The resulting multilayer perfusable microchannels were seeded with endothelial cells so as to mimic a vascular network.^[^ ^176^ ^]^

LbL coupling with microfluidics was started several years ago, and it was primarily used to create patterned surfaces in order to control cell adhesion^[^ ^177^ ^]^ or to drive the high‐throughput assembly of LbL film libraries.^[^ ^178^ ^]^ A recent review by Yola et al. summarized the achievements relating to the polymeric scaffold buildup.^[^ ^179^ ^]^

### 6.2 LbL Film Preparation in Cell Culture Microplates

Recently, technological developments using an automated liquid handling robot have allowed for depositing biomimetic films directly into cell culture microplates,^[^ ^180^ ^]^ and for investigating cell adhesion and differentiation in 96‐well plates, in a parallel and reproducible manner. An adapted method to deposit LbL in microplates was recently proposed, based on automated pipettes.^[^ ^181^ ^]^ This allowed for studying 100 different combinations of LbL films containing CHI and elastin‐like peptides.

Biomimetic coatings, which are optically transparent, are compatible with microplate readers and common optical microscopes. For instance, as a proof‐of‐concept, film‐coated microplates were employed to assess the differentiation of skeletal progenitors into bone cells at high content. C2C12 muscle cells^[^ ^182^ ^]^ and human periosteum‐derived stem cells (hPDSCs)^[^ ^183^ ^]^ were selected as BMP‐responsive cells, with four different physiologically relevant BMPs (BMP‐2, 4, 7, and 9) studied.^[^ ^184^ ^]^ Overall twenty different experimental conditions were screened, including four BMPs and five increasing loading concentrations of BMPs in the films (0, 2.5, 5, 10, and 20 µg mL^−1^), revealing the specific cellular responses to these BMPs. These data revealed that growing stem cells on bioactive films was possible for at least 2 weeks, as was screening stem cell differentiation on bioactive films at high content.

Using such film‐coated microplates, it is now possible to investigate the combined effects of film stiffness and BMPs on stem cell adhesion and differentiation.^[^ ^93^ ^]^ Furthermore, using selected silencing of each of the BMPs and adhesion receptors, the unique role of each receptor regarding cell adhesion can be revealed, as can be their differentiation in terms of BMP‐mediated cell response.

Last but not least, the automated liquid handling deposition can be performed onto micrometer‐sized scaffolds that are deposited inside single wells of a cell culture microplate. This opens the possibility to engineer microscale niches for cancer cell culture, as well as to investigate cancerous processes. Recently, a mini‐pancreatic tumor was created within a miniaturized 3D‐archiectured scaffold coated with the bioactive films.^[^ ^185^ ^]^ The bioactive films of controlled stiffness were either loaded with FN or BMPs. Two human pancreatic cell line behaviors, including PANC1 (immortalized) and PAN092 (patient‐derived cell line), were systematically compared. The results revealed essential differences in cell responses, more particularly in terms of responses to film stiffness and bBMPs. Cell adhesion, spreading, and proliferation for both cell types on soft and stiff films were significantly improved on FN, while BMP2 improved cell adhesion and inhibited proliferation of PANC1 and PAN092 cells on soft films. Cell adhesion and proliferation of PANC1 were enhanced on BMP4, whereas a bipolar effect was observed for PAN092 cells. Importantly, while PAN092 cells were insensitive to BMPs, PANC1 cells exhibited a strong dose‐dependent BMP response, notably for bBMP2.

This new deposit method may be further used for other types of LbL assemblies in order to test several conditions in parallel. It is well adapted for expensive biomolecules and growth factors, and cells like stem cells, since the 96 well plate format enables to use a smaller volume of biomolecules, amount of cells, and culture medium in comparison to other types of plate format (24, 12 well plates). It also opens perspectives for analyses with new optical methods like automated microscopes for high content imaging^[^ ^93^ ^]^ or high resolution microscopes to study cellular processes at the nanometer scale.^[^ ^100^ ^]^ In the future, one may envision that it may be further used to study signaling pathways and test drugs targetting these pathways.

### 6.3 LbL Development Assisted by AI

AI‐derived methodologies, such as machine learning, are now emerging to make the development of new materials faster and cheaper.^[^ ^186^, ^187^ ^]^ This approach is already widely used in many areas, including biomedicine (mostly in the field of genomics^[^ ^188^ ^]^). Machine learning (ML) enables the creation of models that can efficiently learn from real data.^[^ ^189^, ^190^, ^191^ ^]^ It is particularly useful when working on datasets with non‐linear relationships and for pattern recognition in complex systems.^[^ ^192^, ^193^ ^]^ In addition, such models showcase advantages for investigating quantitative structure‐property relationships.^[^ ^194^ ^]^

ML methodologies in materials science is a subject of current interest, with plenty of practical applications. For example, in the photovoltaic cell development, a recent study used high‐throughput synthesis and ML concomitantly to optimize perovskite‐inspired materials.^[^ ^195^ ^]^ Besides the area of materials, ML has also been used to improve established complex systems such as polyelectrolyte membrane fuel cells. Indeed, not only the material properties, but also the design of the fuel cell and operational controls could be analyzed simultaneously.^[^ ^196^ ^]^ Another very concrete and impactful use of ML technologies is in the construction industry, where the incorporation of new components into cement formulations is facilitated by ML methodologies.^[^ ^197^ ^]^ A recent editorial stated the potential uses of ML in materials science, where experimental data can be directly linked with physics‐based simulations to create a positive feedback loop. Moreover, the historically available data can be used to train models to gain new insights and research directions.^[^ ^198^ ^]^ This will contribute to make better use of high throughput screening methodologies with improved data analysis via ML. Moreover, this will enable to match the physics‐based predictive models with the empirical data coming from experiments, to account for the inherent assumptions and confounding effects in real‐life environments for the application of new materials.

However, until recently, these approaches have not been used in the field of LbL films.

#### 6.3.1 Prediction of LbL Thickness Using ML

**Figure 9:**
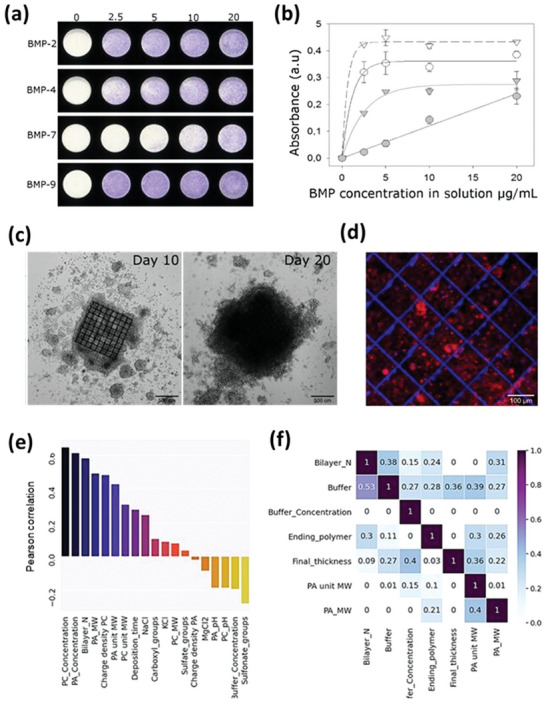
a) Representative images of ALP staing microwells for C2C12 cells culture for 3 days on (PLL/HA)_12_ films built in 96 well plate and post‐loaded with 4 different BMP proteins (BMP‐2, 4, 7, 9) at 5 increasing concentrations of BMP (from 0 to 20 µg mL^−1^), which represents 20 different experimental conditions. b) Absorbance at 570 nm (using a plate reader) representative of ALP expression for each experimental condition. Reproduced with permission.^[^ ^180^ ^]^ Copyright 2018, WILEY‐VCH Verlag GmbH & Co. KGaA, Weinheim. c) 3D culture of pancreatic cancer cells (PANC1) on a bioactive scaffold made of Ormocomp and coated with a crosslinked LbL film made of (PLL/HA) and loaded with fibronectin at 5 µg mL^−1^. Phase contrast images taken after 10 and 20 days of culture. d) After 11 days of culture, the cell cytoskeleton was stained red (rhodamine) and the nucleus blue (DAPI). The scaffold also appears blue. Adapted with permission.^[^ ^185^ ^]^ Copyright 2022, American Chemical Society. e) Pearson correlation and f) predictive power scores were calculated for the final thickness of the coating and coating features. The first seven Pearson correlations are statistically significant (*p* = 0.05). Only features having predictive power scores > 0.001 with the target value are included. Distributed under the terms of the Creative Commons CC BY license. Copyright 2021, The Author(s).^[^ ^200^ ^]^

The development of LbL films with desired properties is a long and costly process, requiring adjustment of multiple parameters, including polymer type, concentration, number of bilayers, and many others. Despite the constantly increasing number of publications in the LbL field,^[^ ^199^ ^]^ no predictive model has been developed so far. Such model would enable us to link polymer‐related parameters, buildup conditions, and properties of the resulting LbL films. One of the reasons for this gap is the absence of appropriate databases. On the other hand, producing a large amount of experimental in‐house data is likewise problematic, given that this would require a lot of time, while being highly expensive. To overcome these limitations, researchers have attempted to extract LbL film buildup parameters from publications, adding them to in‐house produced data.^[^ ^200^ ^]^ LbL film thickness was selected as an output parameter, because it is often measured by researchers in different studies. The resulting dataset was heterogeneous in terms of techniques used for thickness measurement, and it contained multiple missing values. Nevertheless, this work not only allowed to develop predictive models,^[^ ^200^ ^]^ but also to analyze the relative impact of different parameters on LbL film thickness (**Figure** **9**). For instance, the correlations between different variables and coating thickness were evaluated using Pearson's correlation coefficient (for linear relationships) and the predictive power scores method (for non‐linear relationships). The results showed a strong positive linear relationship between the polymer concentration and bilayer number within the coating and resulting thickness (correlation coefficients close to 0.6, Figure 9A), which is quite intuitive. Conversely, polyanion molecular weight and buffer properties displayed non‐linear relationships with coating thickness (Figure 9B).

In a more recent study, missing data imputation allowed for working on a total of 131 instances of coating thickness (extracted from the literature and produced in‐house). As a result, new models permitting better prediction were developed.^[^ ^201^ ^]^ The extra‐tree regression algorithm was the most efficient for predicting LbL coating thickness, the predicted values being close to actual values (Figure 7C). In addition, the six best predictors of the coating thickness were identified including polyanion nation, presence of COO^−^ groups, ending polymer, polycation pH, buffer concentration, and bilayer number. These parameters can be used to predict coating thickness, thus avoiding the requirement of measuring multiple parameters, such as in the original dataset including the 22 input features (molecular weight, duration of each layer deposition, charge density, salt concentration, etc.).

#### 6.3.2 Perspectives of ML to Predict Biological Properties of LbL Films

ML approaches can be used to predict the biological properties of LbL films, including cell adhesion, proliferation, and differentiation, as well as antibacterial and anti‐inflammatory properties. To do so, large datasets are needed. They can be produced in‐house by research teams using standardized protocols. In this case, specialists capable of managing such highly interdisciplinary projects including experimental work (biology, chemistry, materials science) along with data sciences, are required. Another possibility is to establish online databases, where researchers from different teams would share their experimental conditions and associated results. An initiative to warranted in order to start such a project, set up the database, and collect and organize the data.

If we take the biomedical use of LbL as an example for such a project, the first step would be to decide six interrelated outputs, possibly involving cell adhesion (such as stem cell adhesion), cell phenotype (such as stem cell differentiation), bacterial response (risk of infection, inherent antimicrobial properties), immune response (such as macrophage response), blood compatibility, as well as biomechanical properties. A big data/ML approach could provide significant insights into the correlations, as well as antagonistic and synergistic effects regarding these outputs. After setting the aims of the outputs, the next step would be to select many input parameters that can be controlled. Experimental methods and protocols must be specified for each experiment and data collection, so that a clean and inter‐usable data set can be generated. Then, the size of the data set required for a reliable model must be determined. Data collection should be carried out in six to eight independent laboratories in order to avoid any laboratory‐, practice‐, or condition‐related confounding effects or biases. Multiple iterations of training with the constant feed of new data will likely provide a reliable picture of the LbL construction parameters, along with their impact on cells.

By providing new insights, machine learning may revolutionize the design and buildup of LbL films by predicting the conditions needed for a desired function, or at least restraining the number of conditions to be tested. Thus, the ML methodology can be used to provide a faster response to actual and urgent biomedical problems, including antibiotic resistance, organ failure, drug delivery, implant rejection, viral outbreaks, and expedited implementation of new biomedical technologies.

LbL technology has proven easy to combine with other existing methodologies, including microfluids, 3D printing, and high content imaging. The range of possibilities seems almost endless.

## 7 Conclusions and Perspectives

The LbL technology is a versatile tool for biomolecules/drug delivery and tissue engineering, because polymers and biopolymers, including ECM components, proteins, peptides, and growth factors can be used as components of the films. Depending on their physical properties, they can be either directly used to build the films, incorporated into the films, or grafted to one of the polymers constituting the films.

During the past 20 years, new applications have emerged, and LbL films have begun to be used in vitro as models for human tissues and in vivo to repair tissues, with proof‐of‐concept in small and large animal models. Applications are now close to a clinical use, which may be achieved in the next few years.

There are almost endless possibilities to gain different functions by varying polymers, deposition conditions, and addition of bioactive molecules. The “mild” conditions used for film buildup, notably the compatibility with aqueous media, are a great advantage for applications to human tissues, since our tissues contain a large amount of water. Avoiding chemical solvents contributes to decrease the risk of toxicity in contact with human tissues.

The recent technological developments regarding automation of film deposition that enables high content and parallelization studies in multiwell microplates, as well as AI for the prediction of film properties and performance, opened new opportunities. It is likely that such technologies will become widely used in the next years, enabling more data to be obtained simultaneously, thanks to the parallelization capacities offered by the multiwell cell culture plate format. Automation should open new avenues for real‐world applications and future commercialization of LbL‐based products. In addition, the desired architecture may be better predicted using in silico methods. For AI, there is an urgent need to create large databases, so that reliable AI models can be developed. This will require a joint effort from all members of the LbL community.

The combination and integration of the LbL assembly with other nano‐ and micro‐technologies would enable a better understanding and characterization of LbL films, eventually accelerating the deposition process while maintaining its flexibility. LbL assembly onto supporting materials or platforms with even more convoluted geometries should accelerate the design, development, and testing of a wide variety of more versatile and complex biofunctional ECM‐mimetic materials exhibiting unique properties and functions.

Devices may be fabricated “on‐demand” and customized, depending on the intended use. It is likely that devices that more closely recreate the in vivo biological microenvironments and the intrinsic features of living systems, including adaptive behaviors, bioinstructive properties, self‐healing ability, and multi‐scale organization will emerge in the field of tissue engineering and regenerative medicine. For instance, healthy or pathological human tissue models, like cancer, will be engineered and may be used to select cells based on their repertoire of cell surface receptors and/or to test drugs.

Issues persist concerning the industrial scalability of the LbL process, notably the amount of raw materials needed, in particular when assembling expensive biological materials, the quality of these raw materials, and the time needed to process the LbL films, mainly when resorting to the most commonly used dip‐coating methodology. The development of new production methods of bioactive molecules, biopolymers, and polymers, offered by the pharmaceutical and chemical industries, will undeniably benefit the LbL field. Indeed, the automatization may enable to test different batches to quantify batch‐to‐batch variability, compare natural‐origin biopolymers, and study the effect of temperature, to name just a few. In the near future, some assembly methodologies may be easier to scale up than others. The need for appropriate production settings, like clean rooms and appropriate sterilization procedures, which need to be compatible with the bioactive compounds such as proteins and peptides, will have to be carefully established.

Last but not least, there is still room for a fundamental understanding of LbL systems, especially those assembled by non‐electrostatic interactions and those providing multi‐functionalities. Here, studies combining experimental data with computational simulations should provide new data. There is still a need to better understand the dynamic mechanisms and properties of LbL films at the molecular scale. Notably, in view of their poor electronic contrast, high water content, and organic composition, biopolymer‐based LbL films and cells are particularly difficult to study using “conventional” materials analysis techniques, like electron‐based or X‐ray‐based techniques. In addition, the very small size of the LbL films, often in the nanometer range, further hampers imaging using standard imaging techniques, which are not sufficiently sensitive at the nanoscale.

Finally, studies need to be performed in physiological environments, including media such as blood, other physiological fluids, and pre‐clinical models, to assess the film stability and biocompatibility in view of future applications in vivo.

## Conflict of Interest

N.E.V. and P.L. are employed by SPARTHA Medical, which is a coating development company. The article does not contain any information about SPARTHA products.
